# A Bioorthogonal TCO–Tetrazine-Based Pretargeted PET/NIRF Platform Enabling High-Contrast Tumor Imaging

**DOI:** 10.3390/ph18121874

**Published:** 2025-12-09

**Authors:** Mingxing Huang, Weichen Wang, Qiao Yu, Yike Zhou, Yingwei Wang, Rang Wang, Xin Li, Yaojia Zhou, Yi Zhang, Rong Tian

**Affiliations:** 1 Department of Nuclear Medicine, West China Hospital, Sichuan University, Chengdu 610017, China; 2 Clinical Nuclear Medicine Research Laboratory, Department of Nuclear Medicine, West China Hospital, Sichuan University, Chengdu 610017, China; 3 Animal Experimental Center of West China Hospital, Sichuan University, Chengdu 610041, China; 4 Department of Biomedical Sciences, Cedars-Sinai Medical Center, Los Angeles, CA 90048, USA

**Keywords:** pretargeting, PET imaging, bioorthogonal chemistry, radiopharmaceuticals

## Abstract

**Objectives**: Pretargeting strategies enhance the specificity and safety of radiopharmaceuticals by separating tumor targeting from radionuclide delivery. To address the rapid clearance and systemic exposure of directly labeled small-molecule agents, a DZ-1–based pretargeting system was developed, utilizing its broad-spectrum tumor-targeting characteristics. **Methods:** Three DZ-TCO precursors (DZ-1-TCO, DZ-Lys-TCO, and DZ-Lys-PEG_4_-TCO) were synthesized and evaluated by near-infrared fluorescence imaging in HeLa and U87MG tumor-bearing mice. Two tetrazine probes (methyl-tetrazine and mono-substituted tetrazine) were labeled with ^68^Ga to yield ^68^Ga-DOTA-Me-Tz and ^68^Ga-DOTA-H-Tz, whose stability was assessed in PBS and serum. Pretargeted PET imaging was performed using different precursor/probe combinations and pretargeting intervals (24, 48, and 72 h). **Results:** All precursors exhibited tumor accumulation peaking at 24 h and signal retention up to 96 h. Both ^68^Ga-DOTA-Me-Tz and ^68^Ga-DOTA-H-Tz maintained >85% radiochemical stability after 4 h. PET imaging identified DZ-Lys-TCO as the most effective precursor (1.98 ± 0.72 %ID/g, T/M 3.86 ± 0.91). Using ^68^Ga-DOTA-H-Tz, the 48 h interval achieved optimal uptake (3.24 ± 0.95 %ID/g) with the highest tumor-to-muscle ratio (8.30 ± 3.39). Biodistribution confirmed rapid renal clearance, low off-target accumulation, and peak tumor uptake of 3.53 ± 1.76 %ID/g (T/M 10.9 ± 0.3 at 30 min). **Conclusions:** The DZ-TCO/^68^Ga-DOTA-Tz pretargeting system enables high-contrast tumor imaging with low background. The combination of DZ-Lys-TCO and ^68^Ga-DOTA-H-Tz at a 48 h interval provides optimal performance, representing a promising platform for precise and safe radiopharmaceutical imaging.

## 1. Introduction

Radiotheranostics has emerged as a promising strategy in precision oncology by enabling tumor visualization and therapy through identical or closely related molecular entities [1,2]. However, directly labeled radiopharmaceuticals often suffer from prolonged circulation, non-specific background uptake, and increased radiation exposure to healthy tissues, thereby limiting their clinical applications, especially for isotopes with long half-lives such as ^177^Lu and ^225^Ac [2,3,4,5]. To overcome these limitations, pretargeting approaches have been increasingly explored as a means to decouple the tumor-targeting moiety from the radioactive payload, thereby improving imaging contrast and therapeutic indices [6,7].

The trans-cyclooctene (TCO)-tetrazine (Tz) inverse electron-demand Diels-Alder (IEDDA) reaction has become one of the most efficient bioorthogonal click chemistry pairs for in vivo applications due to its high specificity, fast reaction kinetics, and biocompatibility [8,9]. Pretargeted PET imaging using TCO-Tz systems allows for rapid clearance of unbound radiotracers and high tumor-to-background contrast, especially when paired with short-lived isotopes like ^68^Ga, ^18^F, or ^64^Cu [10,11,12]. Several studies have demonstrated the clinical potential of TCO-Tz-based pretargeting in antibody- or peptide-mediated tumor imaging [13,14,15], but further advances are needed in the development of versatile, small-molecule-based pretargeting agents that offer improved tumor penetration and rapid systemic clearance.

DZ-1 is a near-infrared (NIR) fluorescent dye with excellent in vivo tumor selectivity across multiple tumor types, including glioma, breast, and hepatocellular carcinoma [16,17,18]. Its low molecular weight, favorable biodistribution, and high tumor uptake make it a suitable candidate for conjugation in theranostic systems. However, direct radiolabeling of DZ-1-based compounds with therapeutic radionuclides has been associated with prolonged systemic exposure and non-target tissue uptake, raising concerns over off-target toxicity [18,19]. To address these limitations, we designed a DZ-1-based pretargeting platform that integrates the DZ scaffold with TCO moieties, enabling bioorthogonal reaction with ^68^Ga-labeled Tz tracers for PET imaging applications.

In this study, we report the synthesis and characterization of a series of DZ-1-TCO derivatives, including DZ-1-TCO, DZ-Lys-TCO, and DZ-Lys-PEG_4_-TCO. These compounds were evaluated for their in vivo tumor-targeting properties via NIR fluorescence imaging, followed by pretargeted PET imaging using ^68^Ga-labeled tetrazine tracers. Additionally, the reaction efficiency, radiolabeling yield, and in vitro stability of ^68^Ga-DOTA-Tz probes were assessed. This study provides proof-of-concept for the application of small-molecule-based pretargeting strategies using DZ-1 derivatives, potentially enabling high-contrast imaging with improved safety profiles.

## 2. Results

### 2.1. Radiochemical Purity and Stability

**Figure 1:**
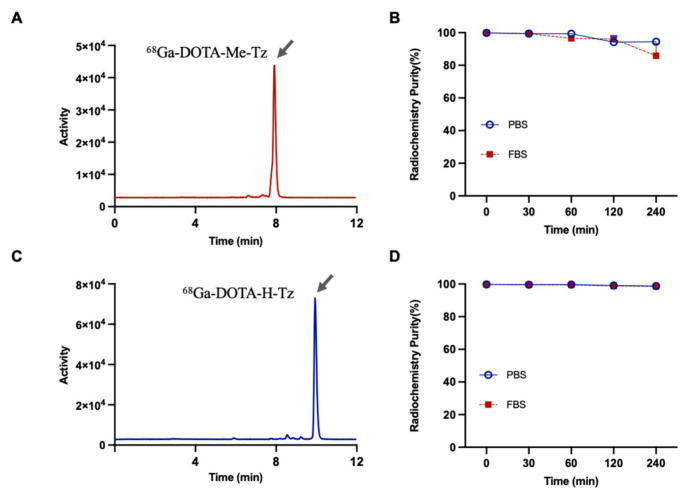
(**A**) Radiochemical purity of ^68^Ga-DOTA-Me-Tz analyzed by radio-HPLC. (**B**) Stability of ^68^Ga-DOTA-Me-Tz in PBS and FBS over time measured by radio-TLC. (**C**) Radiochemical purity of ^68^Ga-DOTA-H-Tz analyzed by radio-HPLC. (**D**) Stability of ^68^Ga-DOTA-H-Tz in PBS and FBS over time measured by radio-TLC.

Two DOTA-conjugated tetrazine derivatives, DOTA-Me-Tz and DOTA-H-Tz, were successfully radiolabeled with ^68^Ga at 95 °C for 15 min. The resulting radiotracers, ^68^Ga-DOTA-Me-Tz and ^68^Ga-DOTA-H-Tz, demonstrated high radiochemical purities, both exceeding 98% as determined by radio-HPLC (Figure 1A,C). The log *p* values were −2.87 ± 0.02 for ^68^Ga-DOTA-Me-Tz and −3.57 ± 0.21 for ^68^Ga-DOTA-H-Tz, indicating high hydrophilicity of both probes.

The initial radiolabeling yields were 99.86 ± 0.11% and 99.77 ± 0.02%, respectively. In vitro stability was assessed in 50% fetal bovine serum (FBS) and 50% phosphate-buffered saline (PBS) at 37 °C for up to 4 h. In FBS, the radiochemical purity of ^68^Ga-DOTA-Me-Tz decreased to 85.88 ± 7.55% at 4 h (Figure 1B), whereas ^68^Ga-DOTA-H-Tz retained higher stability, with 98.53 ± 0.58% remaining intact (Figure 1D). In PBS, both tracers exhibited improved stability, with ^68^Ga-DOTA-Me-Tz and ^68^Ga-DOTA-H-Tz showing radiochemical purities of 94.48 ± 1.48% and 98.76 ± 0.52%, respectively, after 4 h of incubation.

### 2.2. In Vivo Tumor Accumulation and Fluorescence Imaging Outcomes

To evaluate the tumor-targeting characteristics of DZ-TCO derivatives, DZ-1-TCO, DZ-Lys-TCO, and DZ-Lys-PEG_4_-TCO were administered to HeLa and U87MG xenograft-bearing nude mice (100 μL of 50 μM, *n* = 3 per group). In vivo fluorescence imaging was conducted at 4, 24, 48, 72, and 96 h after TCO compound injection.

**Figure 2:**
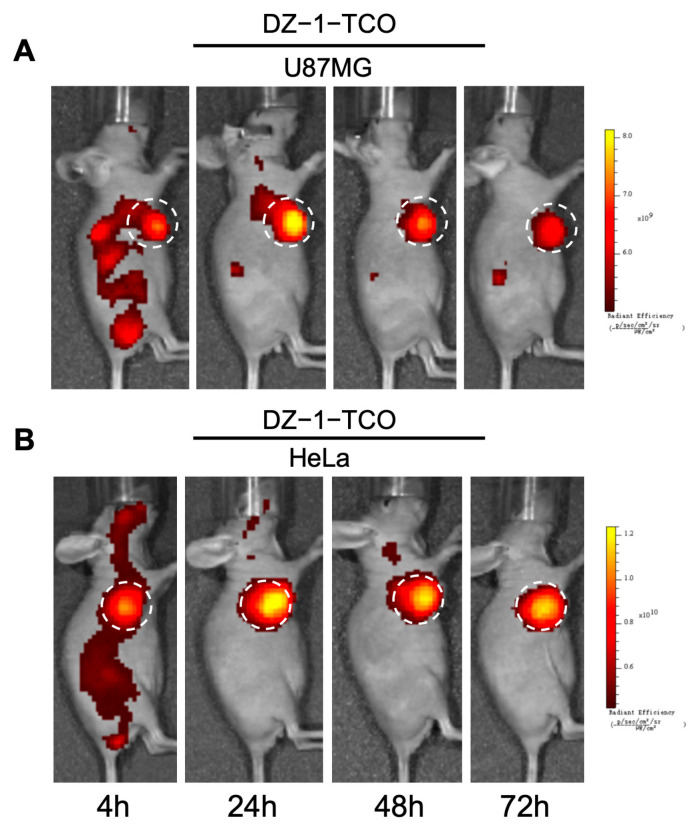
Fluorescence images of U87MG (**A**) and HeLa (**B**) tumor models after intravenous injection of DZ-1-TCO. White dashed circles indicate the tumor regions.

DZ-1-TCO exhibited visible fluorescence accumulation at tumor sites from 4 h post-injection, with intensities of 6.38 × 10^9^ p/s/cm^2^/sr in HeLa and 5.44 × 10^9^ p/s/cm^2^/sr in U87MG tumors. The signal peaked at 24 h (6.93 × 10^9^ and 6.12 × 10^9^ p/s/cm^2^/sr, respectively), declined at 48 h (5.57 × 10^9^ in HeLa and 4.82 × 10^9^ in U87MG), and remained detectable at 72 h. Fluorescence in the liver was consistently higher than in the kidneys across all time points (Figure 2 and Figure S2).

**Figure 3:**
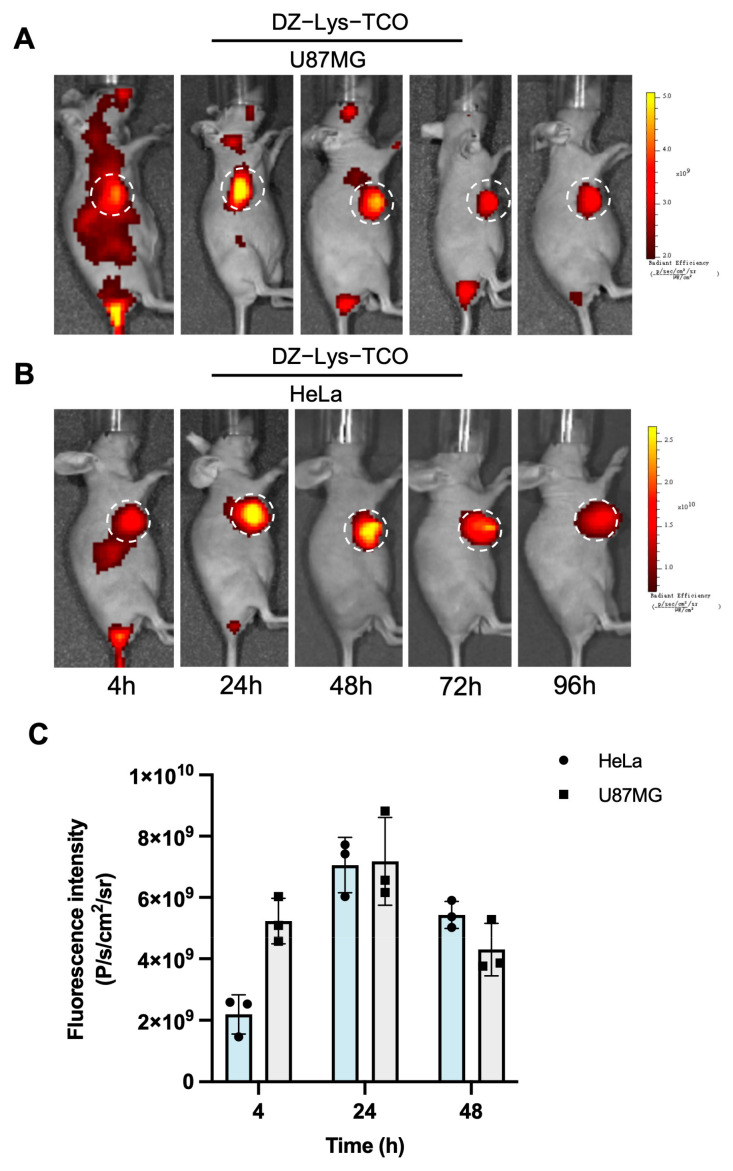
(**A**) In vivo fluorescence imaging of DZ-Lys-TCO in U87MG tumor-bearing mice. (**B**) In vivo fluorescence imaging of DZ-Lys-TCO in HeLa tumor-bearing mice. (**C**) Semi-quantitative analysis of fluorescence intensity in tumor regions. White dashed circles indicate tumor locations.

DZ-Lys-TCO was rapidly distributed throughout the body and progressively accumulated in tumors. Fluorescence intensities at the tumor sites were 2.19 × 10^9^ p/s/cm^2^/sr (HeLa) and 5.23 × 10^9^ p/s/cm^2^/sr (U87MG) at 4 h post-injection, increasing to 7.06 × 10^9^ and 7.18 × 10^9^ p/s/cm^2^/sr at 24 h, respectively. Elevated signals persisted at 48 h (5.43 × 10^9^ in HeLa; 4.30 × 10^9^ in U87MG) and remained detectable up to 168 h (Figure 3). Fluorescence in non-target organs was relatively low, with signal intensities of 8.77 × 10^7^ (spleen), 2.02 × 10^8^ (lungs), 5.04 × 10^8^ (liver), and 3.95 × 10^8^ p/s/cm^2^/sr (kidneys) at 48 h. By 96 h, liver and spleen signals declined to 4.38 × 10^8^ and 1.24 × 10^8^ p/s/cm^2^/sr, respectively, and fluorescence in normal tissues was nearly undetectable at 168 h (Figure S3).

**Figure 4:**
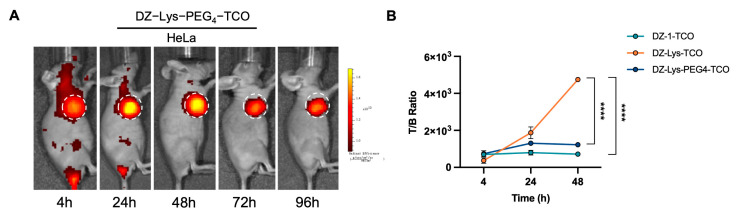
(**A**) In vivo fluorescence imaging of DZ-Lys-PEG_4_-TCO in HeLa tumor-bearing mice. (**B**) Tumor-to-background fluorescence intensity ratios of DZ-1-TCO, DZ-Lys-TCO, and DZ-Lys-PEG_4_-TCO (**** *p* < 0.0001). White dashed circles indicate tumor locations.

Intravenously administered DZ-Lys-PEG_4_-TCO exhibited progressive tumor accumulation, peaking at 24 h (1.20 × 10^10^ p/s/cm^2^/sr), declining by 48 h (9.38 × 10^8^ p/s/cm^2^/sr), and remaining detectable at 96 h. Off-target fluorescence was lower than that of the other compounds, with notably reduced hepatic uptake, which is likely attributable to the PEG_4_ linker (Figure 4A and Figure S4).

Quantitative analysis of tumor-to-background fluorescence ratios revealed distinct pharmacokinetic behaviors among DZ-1-TCO, DZ-Lys-TCO, and DZ-Lys-PEG_4_-TCO. DZ-1-TCO produced ratios of 695.00 ± 59.15 at 4 h and 720.00 ± 64.21 at 48 h. For DZ-Lys-PEG_4_-TCO, the ratio was 737.00 ± 159.93 at 4 h, rising to 1303.33 ± 116.76 at 24 h before slightly declining at 48 h. Notably, DZ-Lys-TCO showed a sustained increase, reaching 368.67 ± 151.28 at 4 h and peaking at 4746.67 ± 100.66 at 48 h, significantly exceeding the other compounds (*p* < 0.0001) (Figure 4B).

### 2.3. In Vitro Validation of Bioorthogonal Click Reactivity

The bioorthogonal conjugation of DZ-1-TCO (500 μmol) with DOTA-Me-Tz (10 mmol) was confirmed by LC-MS following a 20 min incubation at room temperature. DZ-1-TCO (C_52_H_69_ClN_4_O_6_S) was detected with a measured *m*/*z* of 456.9 for [M+2H]^2+^, consistent with its theoretical molecular weight of 913.7. DOTA-Me-Tz (C_33_H_51_N_9_O_11_) exhibited a measured *m*/*z* of 750.3 for [M+H]^+^, in close agreement with the theoretical value of 749.8. The product, DZ-1-TCO-Me-Tz-DOTA (C_85_H_118_ClN_11_O_17_S), yielded a measured *m*/*z* of 545.2 for [M+3H]^3+^, corresponding to the theoretical molecular weight of 1633.5 (Figure S5).

### 2.4. In Vivo Pretargeted PET Imaging Performance

#### 2.4.1. Pretargeted PET Imaging Using DZ-TCO Compounds and ^68^Ga-DOTA-Me-Tz

**Figure 5:**
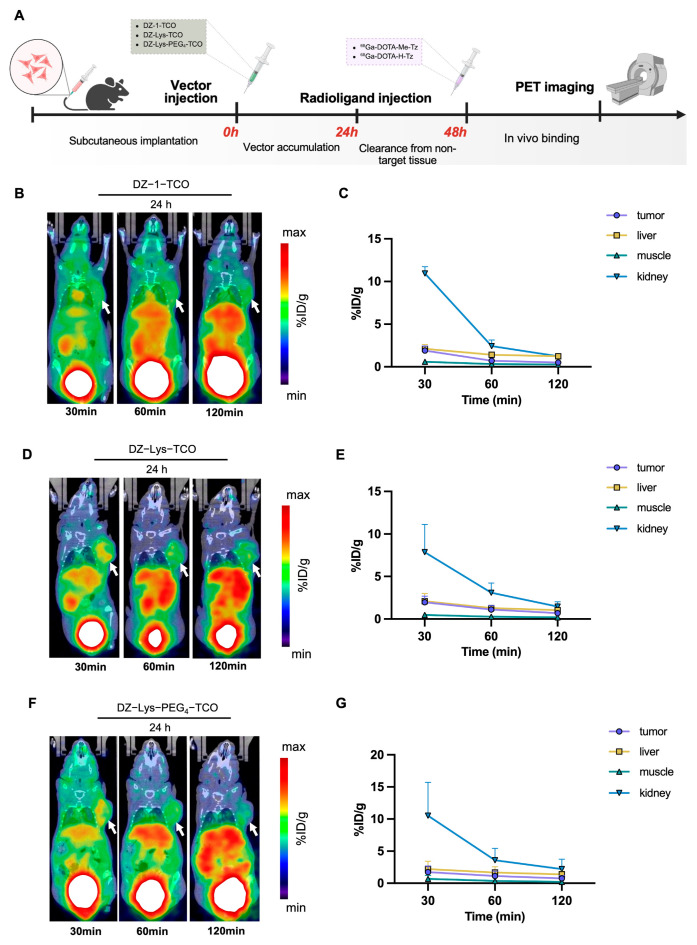
(**A**) Pretargeted imaging administration scheme. (**B**) Representative micro-PET/CT images at different time points after injection of ^68^Ga-DOTA-Me-Tz, following pre-injection of DZ-1-TCO (24 h interval). (**C**) Semi-quantitative analysis of radioactivity uptake (%ID/g) in tumors and major organs for the DZ-1-TCO pretargeting group. (**D**) Representative micro-PET/CT images at different time points after injection of ^68^Ga-DOTA-Me-Tz, following pre-injection of DZ-Lys-TCO (24 h interval). (**E**) Semi-quantitative analysis of radioactivity uptake (%ID/g) in tumors and major organs for the DZ-Lys-TCO pretargeting group. (**F**) Representative micro-PET/CT images at different time points after injection of ^68^Ga-DOTA-Me-Tz, following pre-injection of DZ-Lys-PEG_4_-TCO (24 h interval). (**G**) Semi-quantitative analysis of radioactivity uptake (%ID/g) in tumors and major organs for the DZ-Lys-PEG_4−_TCO pretargeting group. Arrows indicate tumor locations.

In pretargeting PET imaging, mice pre-injected with DZ-1-TCO and administered ^68^Ga-DOTA-Me-Tz after 24 h exhibited rapid tumor accumulation. Uptake peaked at 30 min (1.90 ± 0.54 %ID/g) and decreased to 0.70 ± 0.19 %ID/g at 60 min and 0.50 ± 0.09 %ID/g at 120 min. Prominent bladder activity was observed, indicative of renal-bladder clearance. Liver uptake remained higher than tumor uptake throughout the study (2.12 ± 0.48 %ID/g at 30 min; 1.24 ± 0.13 %ID/g at 120 min), whereas muscle uptake was minimal (0.33 ± 0.06 %ID/g at 60 min) (Figure 5B,C, Table S1).

Pretargeting imaging with DZ-Lys-TCO and ^68^Ga-DOTA-Me-Tz revealed distinct tumor localization, characterized by clear accumulation and sharp tumor-to-background contrast. Tumor uptake reached 1.98 ± 0.72 %ID/g at 30 min, declining to 0.69 ± 0.27 %ID/g at 120 min. Renal-bladder clearance facilitated rapid elimination of unbound tracer, while liver uptake remained slightly higher than tumor uptake, peaking at 2.22 ± 0.76 %ID/g. Relative to DZ-1-TCO, DZ-Lys-TCO yielded lower muscle uptake (0.27 ± 0.11 %ID/g vs. 0.33 ± 0.06 %ID/g) and higher tumor uptake at 60 min (1.10 ± 0.46 %ID/g vs. 0.70 ± 0.19 %ID/g) (Figure 5D, Table S1).

Pretargeting PET imaging with DZ-Lys-PEG_4_-TCO and ^68^Ga-DOTA-Me-Tz demonstrated moderate tumor accumulation, peaking at 30 min (1.73 ± 0.97 %ID/g) and declining to 0.76 ± 0.44 %ID/g at 120 min. The PET images indicated heterogeneous intratumoral distribution of radioactivity. Liver uptake exceeded tumor uptake throughout the study (2.22 ± 1.21 %ID/g at 30 min; 1.40 ± 0.46 %ID/g at 120 min), while muscle uptake remained low and decreased over time (0.68 ± 0.54 %ID/g at 30 min; 0.23 ± 0.04 %ID/g at 120 min) (Figure 5F,G, Table S1).

Quantitative analysis of tumor-to-background ratios revealed distinct patterns among the three compounds. DZ-1-TCO demonstrated a declining trend, with the T/M ratio dropping from 3.08 ± 0.70 at 30 min to 1.75 ± 0.17 at 120 min, and the T/L ratio decreasing from 0.91 ± 0.21 to 0.40 ± 0.09. DZ-Lys-PEG_4_-TCO produced relatively stable values, reaching a maximum T/M ratio of 3.31 ± 1.65 and a maximum T/L ratio of 0.77 ± 0.11. In contrast, DZ-Lys-TCO exhibited a continuous increase, achieving the highest T/M ratio of 3.86 ± 0.91, which was significantly greater than DZ-1-TCO (*p* < 0.05), along with a peak T/L ratio of 0.89 ± 0.29 (Figure S6).

#### 2.4.2. Pretargeted PET Imaging Using DZ-TCO Compounds and ^68^Ga-DOTA-H-Tz

**Figure 6:**
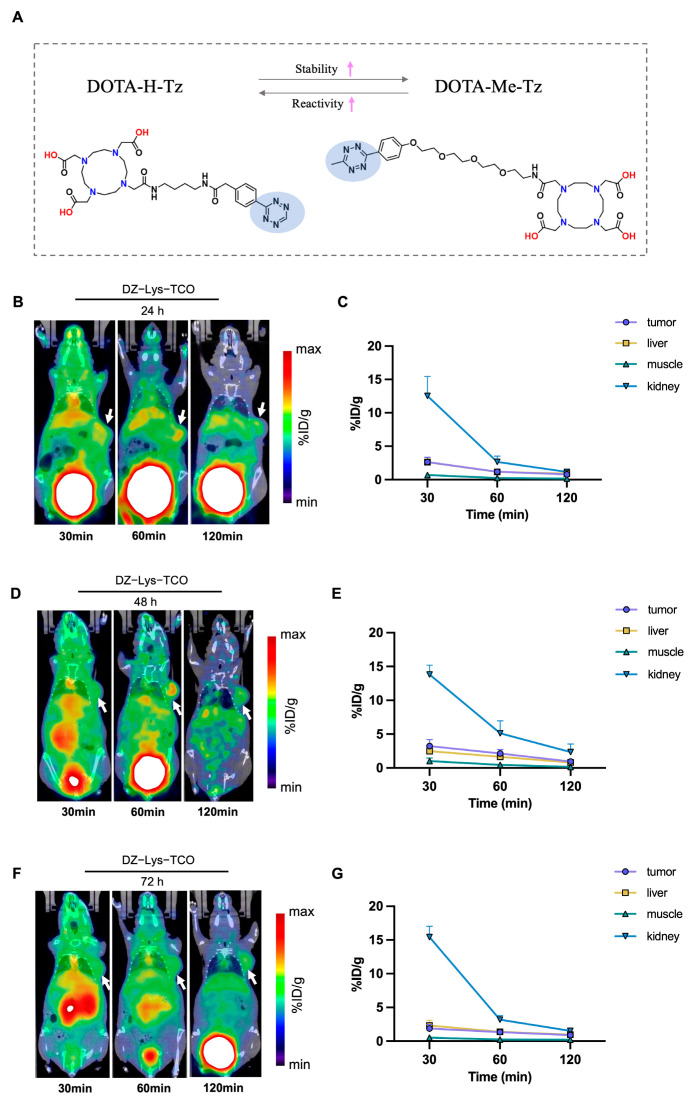
(**A**) Chemical structures of DOTA-Me-Tz and DOTA-H-Tz. The pink arrows indicate the relative increase in stability (↑) and reactivity (↑) between the two tetrazines. (**B**,**D**,**F**) Representative micro-PET/CT images at various time points after injection of ^68^Ga-DOTA-H-Tz following pre-administration of DZ-Lys-TCO at 24 h (**B**), 48 h (**D**), and 72 h (**F**); White arrows highlight tumor sites. (**C**,**E**,**G**) Quantitative biodistribution analysis (%ID/g) in tumor, liver, kidney, and muscle corresponding to the 24 h (**C**), 48 h (**E**), and 72 h (**G**) imaging time points.

To evaluate the effect of using ^68^Ga-DOTA-H-Tz as the secondary agent, micro-PET/CT imaging was performed with different intervals between DZ-Lys-TCO and ^68^Ga-DOTA-H-Tz administration. Pretargeting PET revealed distinct biodistribution patterns depending on the injection interval. With a 24 h interval, rapid renal–bladder clearance of the tracer was evident, and tumors were clearly delineated from surrounding tissues. Tumor uptake was 2.64 ± 0.73 %ID/g at 30 min, decreasing to 1.18 ± 0.25 %ID/g at 60 min and 0.79 ± 0.14 %ID/g at 120 min, while liver uptake was comparable (2.61 ± 0.53, 1.15 ± 0.16, and 0.91 ± 0.16 %ID/g, respectively) (Figure 6B,C). At a 48 h interval, tumor uptake reached 3.24 ± 0.95 %ID/g at 30 min and decreased to 0.96 ± 0.29 %ID/g at 120 min. Uptake remained higher than liver values at all time points, and clearance from non-target tissues at 120 min improved tumor contrast (Figure 6D,E). By contrast, at 72 h, tumor uptake was relatively low (1.89 ± 0.57, 1.35 ± 0.31, and 0.91 ± 0.31 %ID/g at 30, 60, and 120 min, respectively), while liver uptake consistently exceeded tumor uptake (2.32 ± 0.79, 1.40 ± 0.43, and 0.97 ± 0.05 %ID/g) (Figure 6F,G).

Quantitative analysis of tumor-to-organ ratios highlighted differences among the three pretargeting intervals. At 24 h, the T/L ratio at 60 min was 1.01 ± 0.08, and the maximum T/M ratio was 5.31 ± 0.48. At 72 h, tumor contrast improved modestly, with a T/L ratio of 0.98 ± 0.10 at 60 min and a maximum T/M ratio of 5.84 ± 1.49, although absolute tumor signal intensities remained low. In contrast, the 48 h interval provided superior tumor discrimination, with significantly higher ratios of 1.35 ± 0.26 (T/L) and 8.30 ± 3.39 (T/M) (*p* < 0.05) (Figure S7).

### 2.5. Quantitative Biodistribution and Tumor Selectivity Analysis

Following intravenous injection of ^68^Ga-DOTA-H-Tz at a 48 h pretargeting interval, renal clearance was predominant, with kidney uptake decreasing from 10.71 ± 1.34 %ID/g at 30 min to 6.54 ± 1.95 %ID/g at 60 min and 3.28 ± 1.00 %ID/g at 120 min. Liver uptake remained low throughout the study (2.95 ± 1.04, 1.56 ± 0.49, and 0.76 ± 0.23 %ID/g, respectively).

Radioactivity in blood and other non-target organs declined steadily; blood uptake decreased from 2.96 ± 0.11 to 0.64 ± 0.18 %ID/g, and spleen uptake decreased from 1.03 ± 0.08 to 0.24 ± 0.06 %ID/g between 30 and 120 min, with similar trends in muscles and lungs.

**Figure 7:**
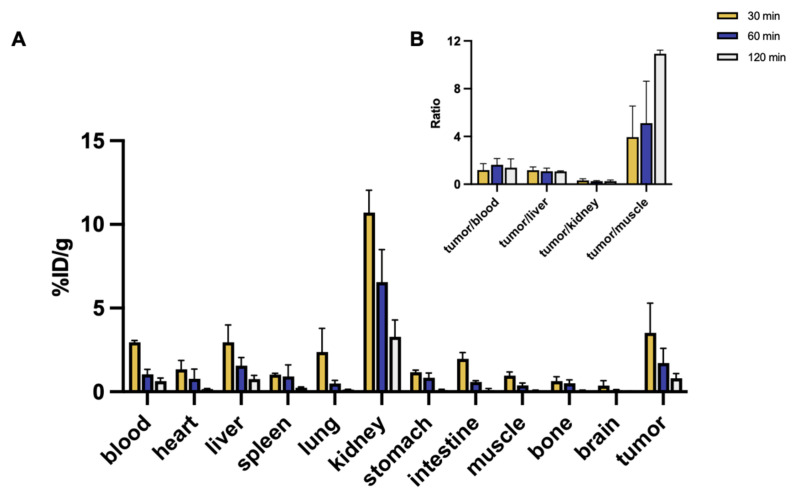
(**A**) Biodistribution of ^68^Ga-DOTA-H-Tz at 48 h post DZ-Lys-TCO pre-injection in HeLa tumor-bearing mice (%ID/g). (**B**) Tumor-to-blood, tumor-to-liver, tumor-to-kidney, and tumor-to-muscle uptake ratios.

Tumor uptake was 3.53 ± 1.76 %ID/g at 30 min, 1.72 ± 0.87 %ID/g at 60 min, and 0.82 ± 0.28 %ID/g at 120 min. Tumor-to-background ratios improved over time, with tumor-to-blood ratios of 1.18 ± 0.55, 1.63 ± 0.53, and 1.39 ± 0.74; tumor-to-liver ratios of 1.18 ± 0.28 (30 min) and 1.07 ± 0.05 (120 min); and tumor-to-muscle ratios rising from 3.95 ± 2.59 to 10.92 ± 0.31. Bone uptake remained consistently low (<0.70 %ID/g) (Figure 7 and Table S2).

## 3. Discussion

Bioorthogonal click-chemistry-based pretargeting has emerged as a promising approach to enhance tumor selectivity and reduce systemic dose in radionuclide imaging and therapy [20,21]. Although radiolabeled DZ-HX derivatives such as ^177^Lu-DZ-HX have demonstrated potent antitumor efficacy in multiple models, their prolonged circulation time may lead to off-target radiation exposure, thus limiting translational applicability [19]. In this study, we established a small-molecule pretargeting platform using a DZ-TCO scaffold in combination with ^68^Ga-labeled tetrazine probes. This design aims to overcome the pharmacokinetic limitations commonly observed in antibody-based pretargeting systems.

Among the tested compounds, DZ-Lys-TCO exhibited the most favorable in vivo characteristics, including high tumor uptake, rapid background clearance, and superior tumor-to-organ contrast. Although aqueous solubility was not experimentally quantified in this work, this trend can be rationalized from a structural perspective. The incorporation of a lysine residue increases local hydrophilicity while minimizing steric shielding around the TCO motif, features that may facilitate systemic circulation and bioorthogonal accessibility. In contrast, the PEG4-modified analogue introduces greater steric bulk and conformational flexibility, which may partially mask the TCO group and is consistent with its comparatively lower tumor uptake [22,23].

In a broader context, small-molecule platforms such as the one reported here offer significant advantages over antibody-based pretargeting systems. For instance, Rossin et al. developed a CC49-TCO/^111^In-DOTA-Tz system that required ≥72 h intervals between vector and probe administration to achieve optimal contrast, with a T/M ratio of 13.1 at 24 h post-probe injection [24]. In the current study, the DZ-Lys-TCO/^68^Ga-DOTA-H-Tz pair achieved comparable tumor-to-muscle ratios (T/M = 10.92 ± 0.31) within a shortened interval of 48 h, highlighting improved imaging flexibility and clinical operability.

The reactivity of the tetrazine partner significantly influences imaging performance. DOTA-H-Tz, a high-reactivity derivative used herein, outperformed the widely used DOTA-Me-Tz in both tumor uptake and image contrast, enabling high signal intensity within 30–60 min post-injection. This aligns with kinetic data indicating that aryl-substituted Tz structures facilitate faster in vivo ligation than methyl-substituted analogs [25]. Notably, DOTA-H-Tz combined with DZ-Lys-TCO achieved higher tumor-to-background ratios than several existing platforms, including the anti-CD11b-TCO/^68^Ga-NOTA-polypeptide-Tz system reported by Zhang et al. (T/M = 5.94 ± 1.46 at 48 h) [26].

Mechanistically, the prolonged tumor retention of DZ-Lys-TCO may partially derive from albumin binding via the meso-chlorine site of the DZ core. This feature has been shown to enhance serum half-life and promote EPR-mediated tumor accumulation, thereby providing a broader window for Tz administration [27,28]. Such pharmacokinetics are particularly advantageous for small-molecule pretargeting systems, enabling flexible probe scheduling without compromising specificity.

The biodistribution studies further supported the translational potential of the DZ-Lys-TCO system. Post-injection of ^68^Ga-DOTA-H-Tz, high tumor uptake and rapid non-specific clearance were observed, with tumor-to-blood and tumor-to-liver ratios favorable for both diagnostic and potential therapeutic applications. Compared to antibody-TCO platforms that often require dose optimization to minimize immunogenicity and background signal [29], the DZ-based approach allows for modular design with minimal systemic burden.

Despite these advantages, several limitations remain. The in vivo stability of the TCO moiety under oxidative and reductive conditions needs further evaluation, as isomerization to cis-cyclooctene may reduce click efficiency. Additionally, while the imaging potential has been well characterized, therapeutic validation using radiotherapeutic isotopes such as ^177^Lu or ^161^Tb is needed to confirm the utility of this platform in targeted radionuclide therapy. Future work should also explore combination strategies with fractionated dosing, immune modulation, or dual-isotope regimens to further enhance therapeutic efficacy.

## 4. Materials and Methods

High-quality chemicals and reagents were purchased from standard sources such as Sigma-Aldrich (St. Louis, MO, USA) and Bide Pharmatech Ltd. (Shanghai, China). Deionized water (18.2 MΩ·cm) used for making solutions was obtained from Milli-Q Direct Ultrapure Water System from Millipore (Billerica, MA, USA). DOTA-Me-Tz and DOTA-H-Tz were purchased from Xi’an Kefu Nuobio Technology Co., Ltd. (Xi’an, China). ^68^GaCl_3_ was eluted from the ^68^Ge/^68^Ga generator (Isotope Technologies Garching, Munich, Germany). Liquid chromatography–mass spectrometry (LC-MS) analysis was performed on an Agilent InfinityLab mass spectrometer coupled to an Agilent 1260 Series LC system, equipped with an Eclipse Plus C18 column (4.6 × 100 mm, 3.5 µm) (all from Agilent Technologies, Santa Clara, CA, USA), and monitored at 254 nm. Radiochemical purity was assessed by radio-thin-layer chromatography (rTLC) and reversed-phase high-performance liquid chromatography (RP-HPLC) using a Kinetex C18 column (250 × 4.6 mm, 5 µm, Phenomenex, Torrance, CA, USA) with an inline γ-detector (Beijing PET Technology, Beijing, China). The gradient composition and flow rate of the water/0.1% TFA/acetonitrile mobile phase were optimized for each product.

All synthesized compounds were characterized by ^1^H NMR spectroscopy (400 MHz, Bruker Avance II, Bruker, Billerica, MA, USA) and mass spectrometry, and their purity was assessed by RP-HPLC.

### 4.1. Synthesis of DZ-TCO Conjugates

**Scheme 1:**
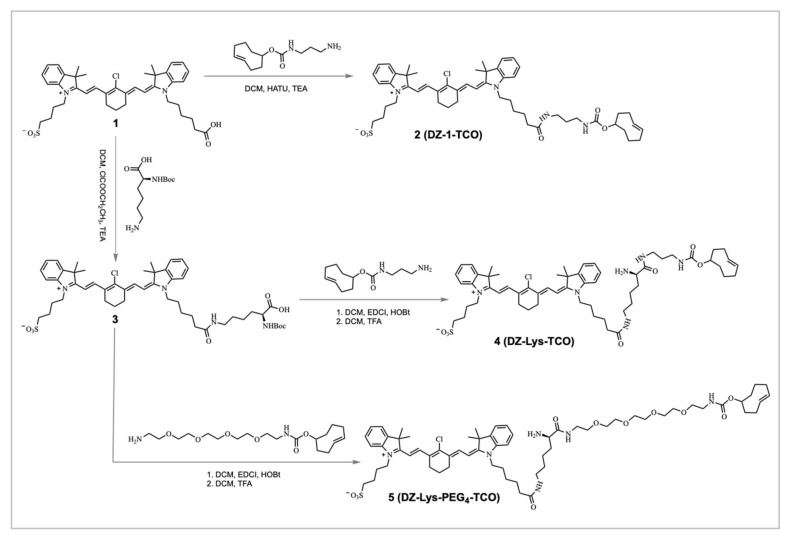
Synthetic routes of DZ-TCO conjugates.

The synthetic routes of the DZ-TCO conjugates are illustrated in Scheme 1.

#### 4.1.1. Synthesis of Compound **2** (DZ-1-TCO)

DZ-1 (1.1 equiv.) and HATU (1.2 equiv.) were dissolved in anhydrous DCM (2 mL), followed by the addition of (4E)-cyclooct-4-en-1-amine (1.0 equiv.) and triethylamine (3.0 equiv.). The reaction mixture was stirred at room temperature for 3 h and monitored by TLC (DCM/MeOH = 8:1). After solvent removal under reduced pressure, the crude product was washed with water and extracted with DCM. The combined organic phase was dried over anhydrous Na_2_SO_4_ and concentrated. Purification by silica gel column chromatography afforded DZ-1-TCO as a green solid (18 mg, 49% yield). MS (ESI): *m*/*z* calcd for C_52_H_69_ClN_4_O_6_S [M+H]^+^ = 913.66; found: 913.50.

#### 4.1.2. Synthesis of Compound **4** (DZ-Lys-TCO)

DZ-1 (1.1 equiv.) was coupled with N-Boc-L-lysine (1.0 equiv.) in the presence of HATU (1.2 equiv.) and triethylamine (3.0 equiv.) in DCM (2 mL) at room temperature for 3 h. After work-up and purification, the intermediate was deprotected using TFA/DCM (1:1, *v*/*v*) for 1 h. The resulting amine was reacted with (4E)-cyclooct-4-en-1-amine (1.2 equiv.), HATU (1.2 equiv.), and triethylamine (3.0 equiv.) in DCM (2 mL) for 3 h. Following standard work-up and silica gel chromatography, DZ-Lys-TCO was obtained as a green solid (12 mg, 30% overall yield). MS (ESI): *m*/*z* calcd for C_54_H_72_ClN_5_O_7_S [M+H]^+^ = 956.51; found: 956.39.

#### 4.1.3. Synthesis of Compound **5** (DZ-Lys-PEG_4_-TCO)

To improve hydrophilicity and optimize in vivo pharmacokinetics, a PEG4 spacer was introduced. DZ-Lys-TCO (1.0 equiv.) was reacted with NH_2_-PEG_4_-TCO (1.2 equiv.) in the presence of HATU (1.2 equiv.) and triethylamine (3.0 equiv.) in DCM (2 mL) at room temperature for 3 h. The reaction mixture was washed, extracted, and purified by silica gel column chromatography to yield DZ-Lys-PEG_4_-TCO as a green solid (7 mg, 45% yield). MS (ESI): *m*/*z* calcd for C_62_H_86_ClN_6_O_11_S [M+H]^+^ = 1164.59; found: 1164.60.

The ^1^H NMR and high-resolution mass spectra of the final compounds are provided in the (Figure S1).

### 4.2. Radiolabeling of Tetrazine Probes

^68^GaCl_3_ (5 mL, 20 mCi) and DOTA-Tz (50 μg) were mixed in a reaction vial. The pH was adjusted to 4.0–4.5 by adding 1 M sodium acetate, and the mixture was heated at 95 °C for 15 min without further purification. Radiochemical purity was confirmed by RP-HPLC. In vitro stability of ^68^Ga-DOTA-Tz was assessed by incubating aliquots in 50% FBS or 50% PBS at 37 °C for 30 min, 1 h, 2 h, and 4 h. The partition coefficient (log *p*) was determined by mixing 1 mL of n-octanol with 1 mL of PBS containing the tracer, vortexing, and centrifuging at 3000 rpm for 5 min. Samples (0.1 mL) from both the organic and aqueous phases were withdrawn, and their radioactivity was measured using a γ-counter (PerkinElmer Wizard, Shelton, CT, USA).

### 4.3. Cell Culture and Animal Model

HeLa (human cervical carcinoma) and U87MG (human glioblastoma) cell lines were obtained from the Cell Bank of the Chinese Academy of Sciences (Shanghai, China). Cells were cultured in Dulbecco’s Modified Eagle Medium (DMEM; Gibco, Grand Island, NY, USA) supplemented with 10% fetal bovine serum (FBS; Gibco) and 1% penicillin–streptomycin (Gibco) at 37 °C in a humidified incubator with 5% CO_2_.

All animal studies followed the Guidelines for the Care and Use of Laboratory Animals and were approved by the Institutional Animal Care and Use Committee (IACUC) of Sichuan University.

Female BALB/c nude mice (4–6 weeks old; 14–20 g), obtained from Beijing Vital River Laboratory Animal Technology Co., Ltd. (Beijing, China), were subcutaneously inoculated with 5 × 10^6^ HeLa or U87MG cells suspended in 100 μL PBS into the right flank. Tumor growth was monitored every 2–3 days using digital calipers, and experiments were initiated when tumor volumes reached approximately 150–300 mm^3^, calculated using the formula Volume = (length × width^2^)/2.

### 4.4. In Vivo Fluorescence Imaging

To assess the tumor-targeting capability and clearance kinetics of DZ-TCO precursors, 100 µL of each compound (50 μmol in PBS) was administered intravenously via the tail vein. Whole-body fluorescence imaging was performed at predetermined time points (4, 24, 48, 72, and 96 h post-injection) using the IVIS Spectrum imaging system (PerkinElmer, Shelton, CT, USA) (excitation/emission, 745/820 nm) with automatic subtraction of background fluorescence during the acquisition. Fluorescence signals were acquired under standardized exposure settings, and region-of-interest (ROI) analysis was conducted using Living Image software version 4.4 (PerkinElmer) to quantify fluorescence intensity in the tumor and major organs.

### 4.5. Pretargeted PET Imaging

To evaluate in vivo tumor targeting and bioorthogonal reaction kinetics, female BALB/c nude mice bearing subcutaneous tumors were intravenously injected with 50 μmol of each DZ-TCO precursor in phosphate-buffered saline (PBS). At predefined intervals (24, 48, or 72 h post-injection), 100 μmol of ^68^Ga-labeled tetrazine probe (^68^Ga-DOTA-Me-Tz or ^68^Ga-DOTA-H-Tz; 3.7 MBq in PBS) was administered via the tail vein. PET imaging was performed using the IRIS system (Inviscan, Paris, France) at 30 min, 1 h, and 2 h post-injection under 2% isoflurane anesthesia. Image reconstruction and quantitative analysis were conducted using Inveon Research Workplace and OsiriX MD software version 10.0.4. Regions of interest (ROIs) were manually defined over tumors and major organs, and radiotracer uptake was expressed as the percentage of the injected dose per gram of tissue (%ID/g).

### 4.6. Biodistribution Studies

To assess the in vivo biodistribution of the pretargeting system, female BALB/c nude mice bearing HeLa tumors (*n* = 3) were first injected intravenously with DZ-TCO. After 48 h, 0.74 MBq of ^68^Ga-DOTA-H-Tz was administered via the tail vein. At 30, 60, and 120 min post-injection, mice were euthanized, and tissues including blood, tumor, liver, kidney, spleen, heart, lung, muscle, stomach, intestines, brain, and bone were excised, weighed, and counted using a γ-counter (Wizard2, PerkinElmer, Shelton, CT, USA). Radiotracer uptake was expressed as the percentage of injected dose per gram of tissue (%ID/g). Tumor-to-organ uptake ratios were calculated accordingly.

### 4.7. Statistical Analysis

All statistical analyses were performed using GraphPad Prism 9 (GraphPad Software, San Diego, CA, USA). Data are presented as mean ± standard deviation (SD). Comparisons between two groups were conducted using the unpaired Student’s *t*-test. A *p* value < 0.05 was considered statistically significant.

## 5. Conclusions

In conclusion, this study establishes a systematically optimized DZ-TCO-based pretargeted imaging system with favorable pharmacokinetics, excellent tumor selectivity, and improved imaging contrast within clinically relevant timeframes. The DZ-Lys-TCO scaffold offers a versatile small-molecule platform for advancing bioorthogonal nuclear medicine applications. These findings not only demonstrate the feasibility of replacing large antibody-based systems with rationally designed small molecules but also provide a foundation for future theranostic development in pretargeted radionuclide therapy.

## Data Availability

The data presented in this study are available in the article and its Supplementary Materials. Additional raw data, including analytical spectra, imaging files, and quantitative datasets, are available from the corresponding author upon reasonable request.

## Supplementary Materials

The supporting information available at https://www.mdpi.com/article/10.3390/ph18121874/s1 includes high-resolution mass spectra of DZ-1-TCO, DZ-Lys-TCO, and DZ-Lys-PEG_4_-TCO (Figure S1); time-dependent fluorescence imaging and quantitative tumor-signal analyses for each DZ-TCO precursor in tumor-bearing mice (Figures S2–S4); LC-MS characterization of the bioorthogonal reaction between DZ-1-TCO and DOTA-Me-Tz (Figure S5); tumor-to-muscle and tumor-to-liver uptake ratios obtained from pretargeted imaging using different DZ-TCO derivatives with a 24-h interval (Figure S6) and using DZ-Lys-TCO combined with ^68^Ga-DOTA-H-Tz at 24, 48, and 72 h intervals (Figure S7); a comparison of in vivo imaging parameters for the three DZ-TCO derivatives at a 24-h pretargeting interval (Table S1); and biodistribution data for DZ-Lys-TCO and ^68^Ga-DOTA-H-Tz following a 48-h pretargeting interval at multiple time points (Table S2).

## Abbreviations

The following abbreviations are used in this manuscript:

DCM	Dichloromethane
DOTA	1,4,7,10-Tetraazacyclododecane-N,N′,N″,N‴-tetraacetic acid
EPR	Enhanced permeability and retention
HPLC/RP-HPLC	High-performance liquid chromatography/reversed-phase high-performance liquid chromatography
IEDDA	Inverse electron-demand Diels–Alder
FBS	Fetal bovine serum
PBS	Phosphate-buffered saline
PET	Positron emission tomography
rTLC/TLC	Radio thin-layer chromatography/thin-layer chromatography
TCO	Trans-cyclooctene
Tz	Tetrazine
NIR/NIRF	Near-infrared/near-infrared fluorescence
