# Supplementary material for: A Bioorthogonal TCO–Tetrazine-Based Pretargeted PET/NIRF Platform Enabling High-Contrast Tumor Imaging
Source: Pharmaceuticals (Basel). 2025 Dec 9;18(12):1874. doi: 10.3390/ph18121874 (PMC12735951; doi:10.3390/ph18121874)
Supplement: Supplementary file 1 [file pharmaceuticals-18-01874-s001.zip › pharmaceuticals-3967309-supplementary.pdf]

Supplementary Materials

Compound 2 ^{1}H NMR (400 MHz, MeOD) δ 8.43 (dd, *J* = 17.0, 11.0 Hz, 2H), 7.52 (dd, *J* = 7.4, 4.7 Hz, 2H), 7.47 – 7.38 (m, 3H), 7.29 (dt, *J* = 15.1, 8.0 Hz, 3H), 6.43 – 6.23 (m, 2H), 5.62 – 5.32 (m, 2H), 4.32 – 4.13 (m, 5H), 3.64 – 3.57 (m, 3H), 3.25 – 3.12 (m, 4H), 3.10 – 2.99 (m, 2H), 2.93 – 2.86 (m, 2H), 2.74 (d, *J* = 17.2 Hz, 4H), 2.32 – 2.17 (m, 5H), 2.06 – 1.83 (m, 10H), 1.73 (t, *J* = 4.1 Hz, 12H), 1.31 (t, *J* = 7.4 Hz, 4H), 1.18 (t, *J* = 7.1 Hz, 4H).

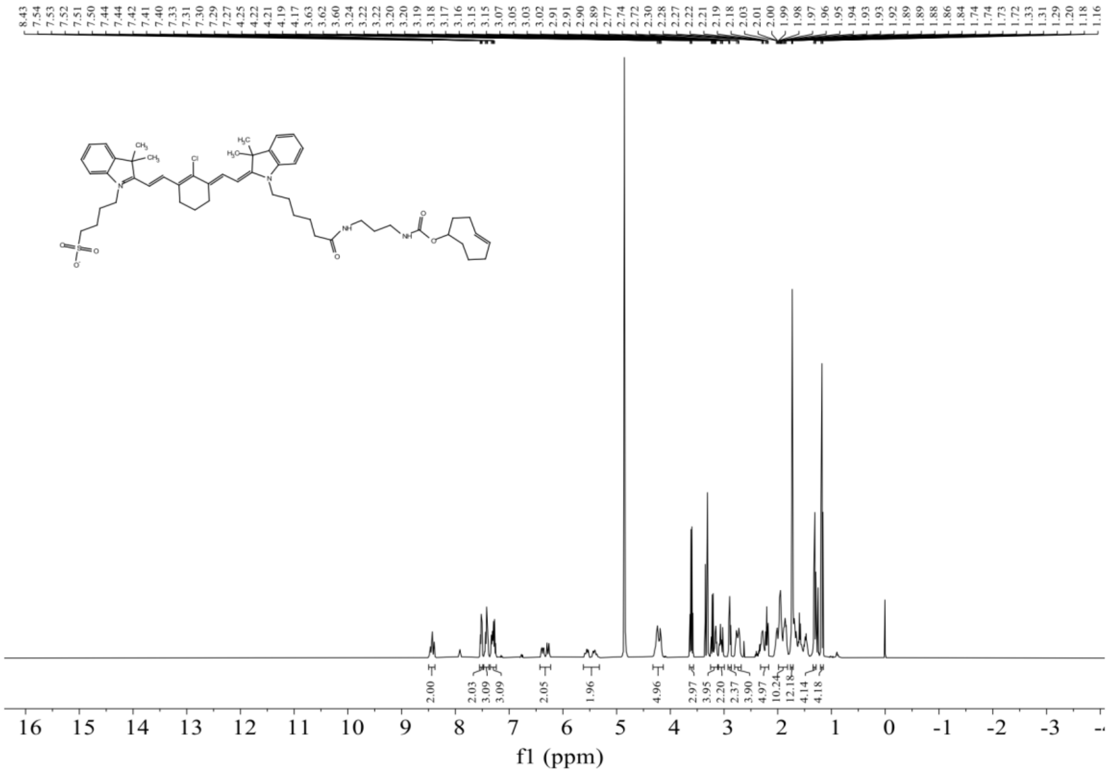

Compound 4 ^{1}H NMR (400 MHz, DMSO-d_6) δ 8.42 (t, *J* = 5.2 Hz, 1H), 8.29-8.20 (m, 2H), 8.08 (d, *J* = 4.0 Hz, 2H), 7.76 (t, *J* = 5.2 Hz, 1H), 7.66-7.61 (m, 2H), 7.53 (d, *J* = 8.0 Hz, 1H), 7.47-7.40 (m, 3H), 7.33-7.24 (m, 2H), 7.03 (t, *J* = 5.6 Hz, 1H), 6.44 (d, *J* = 14.0 Hz, 1H), 6.26 (d, *J* = 14.4 Hz, 1H), 5.70-5.55 (m, 2H), 4.57-4.51 (m, 1H), 4.25 (t, *J* = 6.8 Hz, 2H), 4.18 (t, *J* = 6.8 Hz, 2H), 3.15-3.05 (m, 2H), 3.00-2.95 (m, 4H), 2.74-2.67 (m, 4H), 2.56-2.50 (m, 3H), 2.32-2.05 (m, 1H), 2.12-1.98 (m, 5H), 1.86-1.82 (m, 4H), 1.78-1.70 (m, 6H), 1.67 (d, *J* = 4.0 Hz, 12H), 1.57-1.52 (m, 6H), 1.49-1.41 (m, 2H), 1.36-1.32 (m, 4H), 1.26-1.21 (m, 4H).

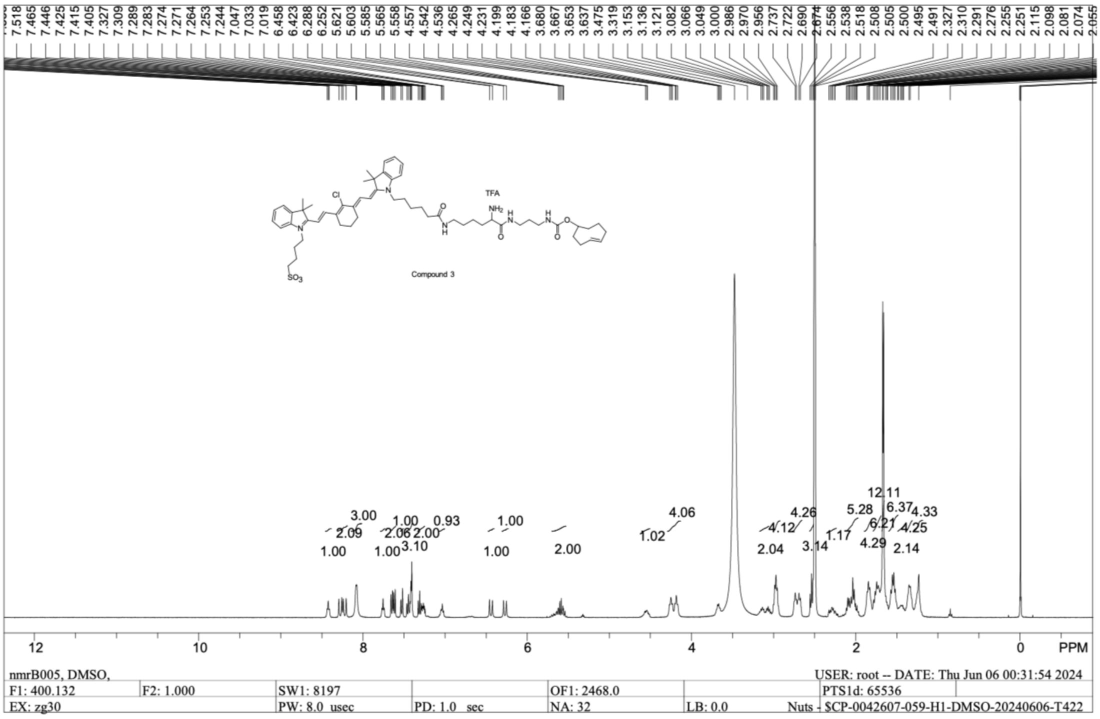

Compound 5 **^{1}H NMR (400 MHz, DMSO-*d₆*)** δ 8.53 (t, *J* = 5.2 Hz, 1H), 8.29-8.20 (m, 2H), 8.08 (d, *J* = 3.6 Hz, 2H), 7.75 (t, *J* = 5.2 Hz, 1H), 7.65-7.60 (m, 2H), 7.52 (d, *J* = 8.0 Hz, 1H), 7.46-7.39 (m, 3H), 7.33-7.24 (m, 2H), 6.98 (t, *J* = 5.6 Hz, 1H), 6.43 (d, *J* = 14.4 Hz, 1H), 6.27 (d, *J* = 14.0 Hz, 1H), 5.71-5.55 (m, 2H), 4.58-4.52 (m, 1H), 4.24 (t, *J* = 6.8 Hz, 2H), 4.18 (t, *J* = 6.4 Hz, 2H), 3.73-3.66 (m, 1H), 3.50-3.46 (m, 12H), 3.42 (s, 2H), 3.38-3.34 (m, 3H), 3.34-3.31 (m, 1H), 3.28-3.24 (m, 1H), 3.10-3.06 (m, 2H), 2.99-2.95 (m, 2H), 2.74-2.67 (m, 4H), 2.55-2.50 (m, 3H), 2.33-2.23 (m, 1H), 2.15-1.98 (m, 5H), 1.86-1.83 (m, 4H), 1.79-1.70 (m, 6H), 1.67 (d, *J* = 4.0 Hz, 12H), 1.63-1.51 (m, 4H), 1.50-1.40 (m, 2H), 1.38-1.31 (m, 4H), 1.28-1.23 (m, 2H).

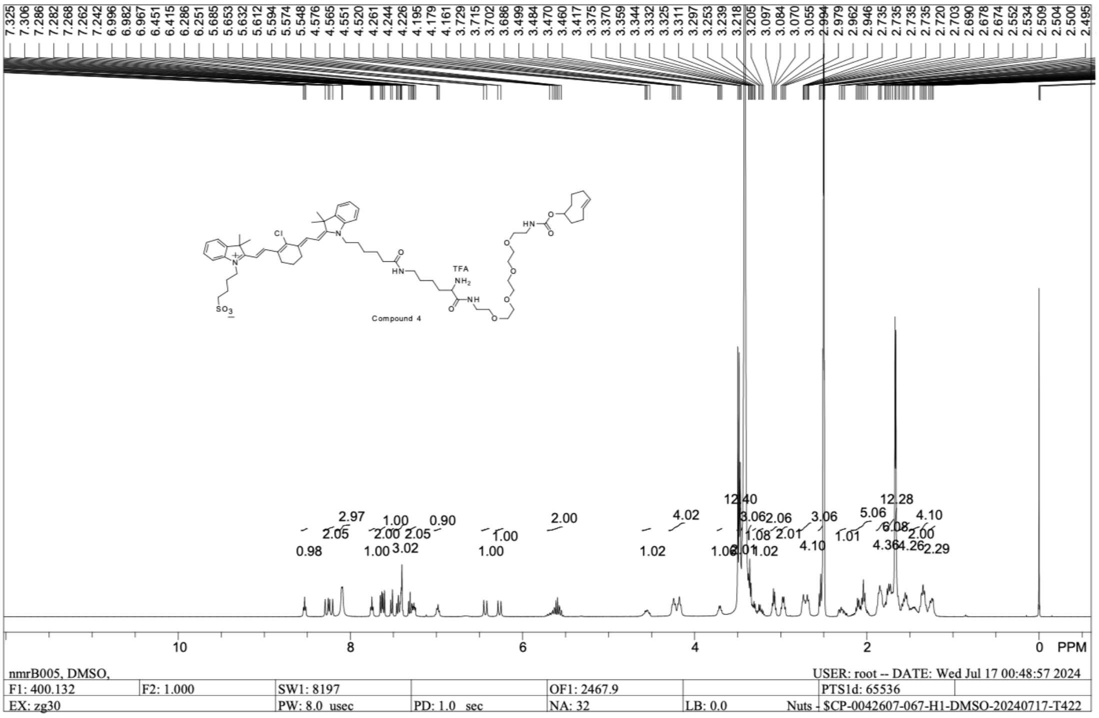

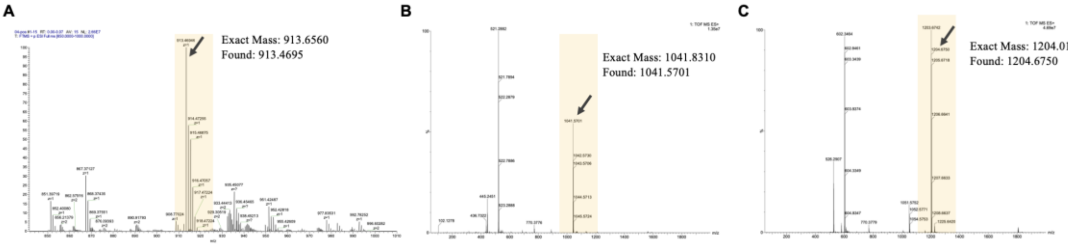

Figure S1. High-resolution mass spectra of the DZ-1-TCO (A), DZ-Lys-TCO (B) and DZ-Lys-PEG_4-TCO (C).

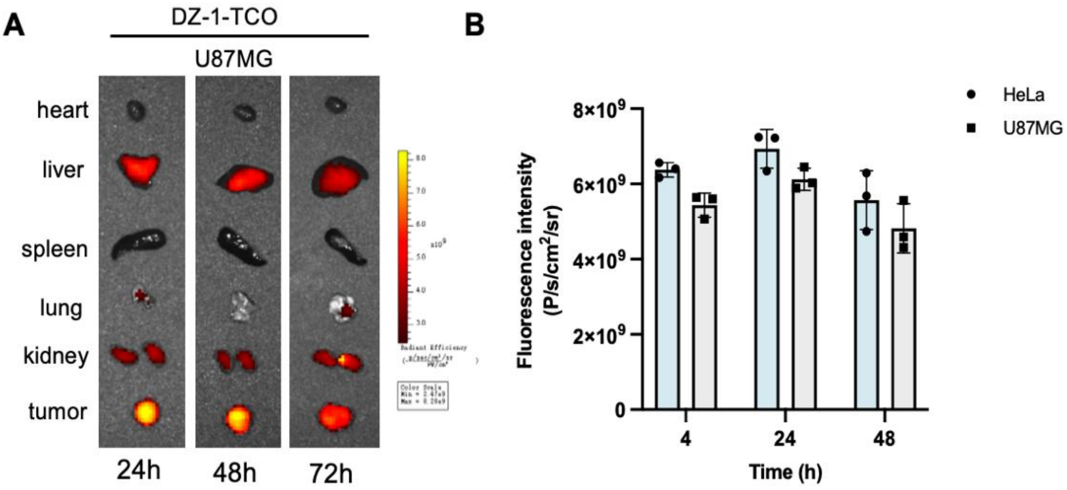

Figure S2. (A) Time-dependent fluorescence imaging of major organs (heart, liver, spleen, lung, kidney) after DZ-1-TCO administration; (B) Semi-quantitative analysis of tumor fluorescence in U87MG and HeLa models.

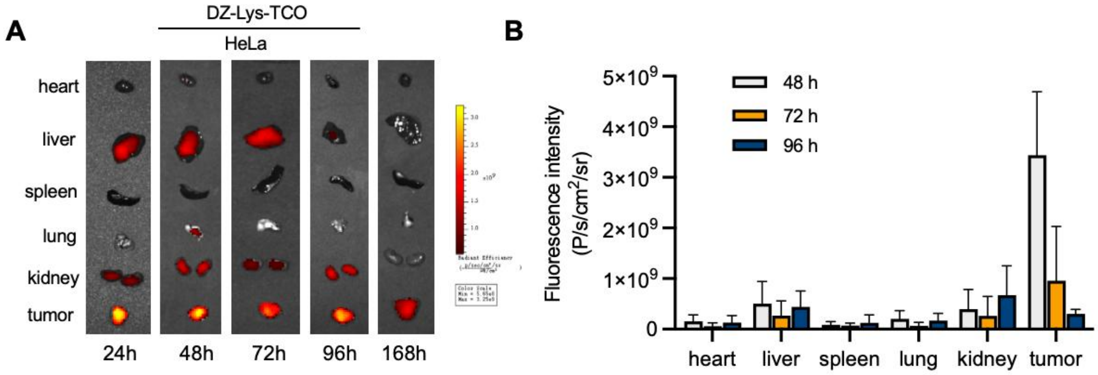

Figure S3. (A) Time-dependent fluorescence imaging of major organs (heart, liver, spleen, lung, kidney) after DZ-Lys-TCO injection; (B) Quantification of fluorescence signals in tumor versus normal tissues.

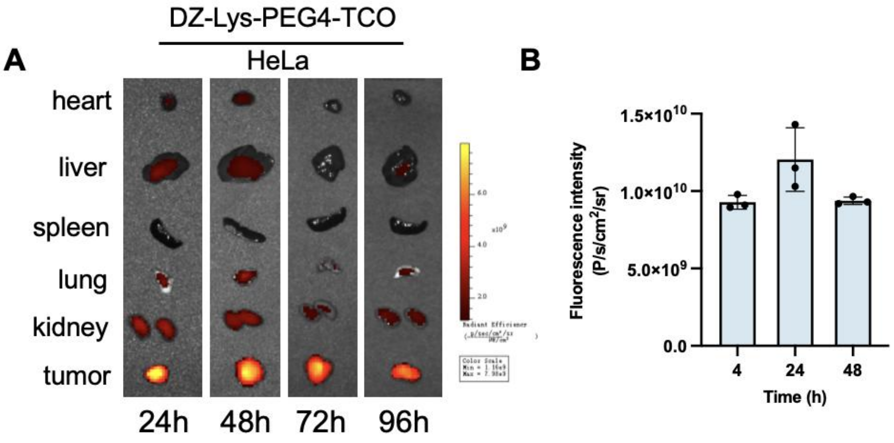

Figure S4. (A) Time-dependent fluorescence imaging of major organs (heart, liver, spleen, lung, kidney) after DZ-Lys-PEG$_4$-TCO injection; (B) Semi-quantitative tumor fluorescence analysis.

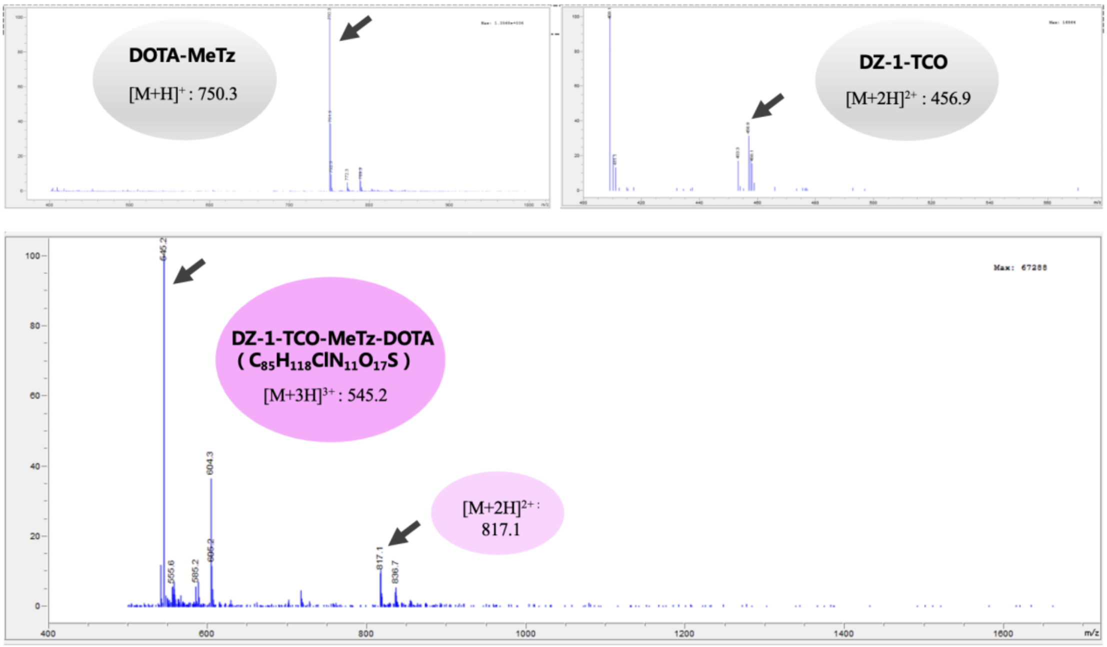

Figure S5. LC-MS analysis of the bioorthogonal reaction between DZ-1-TCO and DOTA-Me-Tz in vitro.

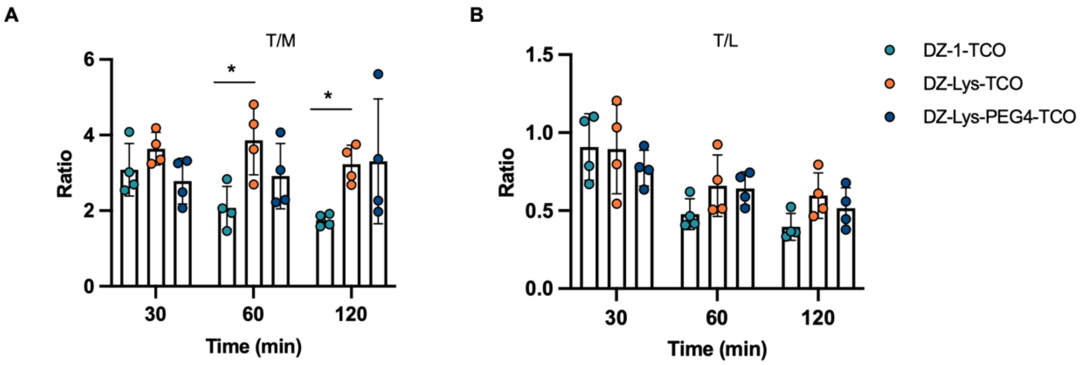

Figure S6. Tumor-to-muscle (A) and tumor-to-liver (B) uptake ratios of DZ-1-TCO, DZ-Lys-TCO, and DZ-Lys-PEG$_4$-TCO with a 24 h pretargeting interval (T = Tumor, M = Muscle, L = Liver, **P* < 0.05).

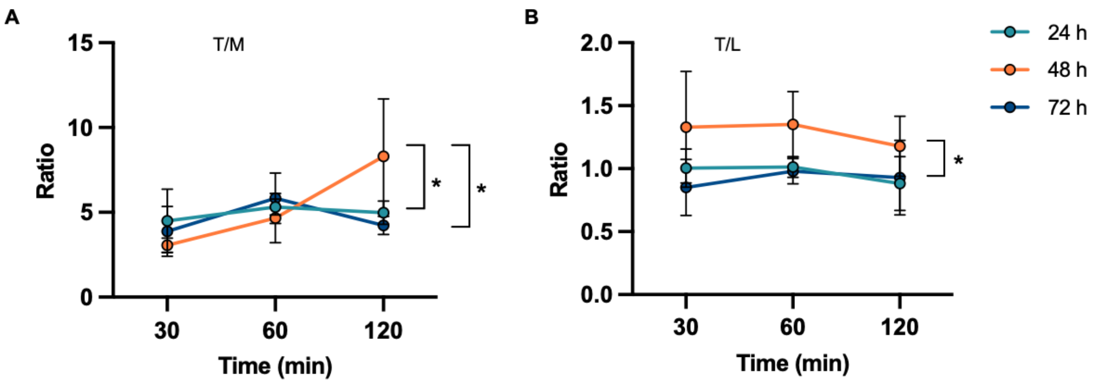

Figure S7. (A) Tumor-to-muscle and (B) tumor-to-liver uptake ratios at 24 h, 48 h, and 72 h after pre-injection of DZ-Lys-TCO, followed by administration of ^{68}Ga-DOTA-H-Tz. (T = Tumor, M = Muscle, L = Liver, **P* < 0.05)

Table S1. Comparative in vivo imaging parameters of DZ-TCO derivatives using a 24-h pretargeting interval*

Parameters	DZ-1-TCO	DZ-Lys-TCO	DZ-Lys-PEG$_4$-TCO
**Peak tumor uptake (%ID/g)**	1.90 ± 0.54	1.98 ± 0.72	1.73 ± 0.97
**Time to peak uptake (min)**	30	30	30
**Peak liver uptake (%ID/g)**	2.12 ± 0.48	2.22 ± 0.76	2.22 ± 1.21
**Peak kidney uptake (%ID/g)**	10.94 ± 0.48	7.85 ± 3.26	10.54 ± 5.17
**Peak muscle uptake (%ID/g)**	0.59 ± 0.19	0.48 ± 0.18	0.68 ± 0.54
**Peak T/M ratio**	3.08 ± 0.70	3.86 ± 0.91	3.31 ± 1.65
**Peak T/L ratio**	0.91 ± 0.21	0.89 ± 0.29	0.77 ± 0.11

*Data are expressed as mean ± SD (%ID/g, n = 3/group). T/M = tumor-to-muscle ratio; T/L = tumor-to-liver ratio. Qualitative interpretation: DZ-1-TCO: Moderate tumor uptake with relatively high kidney background. DZ-Lys-TCO: Highest T/M ratio and lowest kidney uptake, providing the best

tumor-to-background contrast. DZ-Lys-PEG_4-TCO: Lowest tumor uptake and reduced tumor retention compared with the other derivatives.

Table S2. In vivo pretargeting biodistribution of DZ-Lys-TCO and ^{68}Ga-DOTA-H-Tz*

Organ	48 h pretargeting interval		
	30 min	60 min	120 min
**Blood**	2.96 ± 0.11	1.05 ± 0.29	0.64 ± 0.18
**Heart**	1.34 ± 0.53	0.78 ± 0.58	0.17 ± 0.03
**Liver**	2.95 ± 1.04	1.56 ± 0.49	0.76 ± 0.23
**Spleen**	1.03 ± 0.08	0.91 ± 0.70	0.24 ± 0.06
**Lung**	2.38 ± 1.41	0.50 ± 0.18	0.14 ± 0.02
**Kidney**	10.71 ±1.34	6.54 ± 1.95	3.28 ± 1.00
**Stomach**	1.16 ± 0.13	0.83 ± 0.30	0.10 ± 0.05
**Intestine**	1.97 ± 0.38	0.59 ± 0.08	0.11 ± 0.08
**Muscle**	0.97 ± 0.21	0.38 ± 0.15	0.07 ± 0.02
**Bone**	0.64 ± 0.27	0.52 ± 0.20	0.08 ± 0.03
**Brain**	0.37 ± 0.30	0.10 ± 0.03	0.03 ± 0.01
**Tumor**	3.53 ± 1.76	1.72 ± 0.87	0.82 ± 0.28
**T/B**	1.18 ± 0.55	1.63 ± 0.53	1.39 ± 0.74
**T/L**	1.18 ± 0.28	1.08 ± 0.27	1.07 ± 0.05
**T/K**	0.32 ± 0.14	0.25 ± 0.05	0.26 ± 0.10
**T/M**	3.95 ± 2.59	5.11 ± 3.51	10.92 ± 0.31

* All data are presented as %ID/g and expressed as mean ± SD (n = 3/group). T/B = tumor-to-blood ratio; T/L = tumor-to-liver ratio; T/K = tumor-to-kidney ratio; T/M = tumor-to-muscle ratio.
